# Effect of financial incentives on the cost and duration of sickness absence

**DOI:** 10.1371/journal.pone.0305235

**Published:** 2024-06-13

**Authors:** Sheila Timp, Nicky D. van Foreest, Willem van Rhenen

**Affiliations:** 1 Arbo Unie, Groningen, the Netherlands; 2 Faculty of Economics and Business, University of Groningen, Groningen, the Netherlands; 3 Center for Strategy, Organization and Leadership, Nyenrode Business Universiteit, Breukelen, the Netherlands; Universita degli Studi del Piemonte Orientale Amedeo Avogadro, ITALY

## Abstract

Sickness absence is a major concern in public health, affecting individuals, businesses, and society. Developing efficient sickness absence policies could help reduce sickness absence. A key aspect of these policies concerns the financial compensation provided to absent employees, including its amount and the length of time it is offered. This study addresses how financial incentives, like salary reductions, might influence sickness absence. For this purpose, we first develop a model to estimate the sensitivity of employees to a financial incentive using a large dataset consisting of approximately six million sickness cases. We then perform a simulation study to determine the effect of similar incentives at different moments and for varying sensitivities. Our findings indicate that financial incentives can notably shorten the duration of sickness absence and decrease its associated costs, particularly when such incentives are implemented early in the absence period. Incentives implemented later have less impact on absence duration, but can still reduce the overall cost. The results of this study can be used by healthcare professionals and employers in the design and evaluation of diverse sickness absence policies.

## 1 Introduction

In Europe, absenteeism has experienced an increasing trend over the past two decades. Between 2006 and 2020 the sickness absence percentage for women increased from 3.5% to 4.3% and for men from 2.6% to 3.1%. Significant variations in absence rates are observed across European countries, ranging from less than 1% in Iceland, Cyprus, Malta, and Luxembourg, to up to 20% in Germany and France, [1, 2]. As European countries are known for offering some of the most generous sick leave packages in the world, the estimated cost of sick leave is currently 1-2% of GDP (Gross Domestic Product) [3]. As both sickness absence percentages and the associated costs continue to rise, the impact and effectiveness of disability policies become critical in many industrialized countries.

In most countries, benefits are provided for sick employees, but the specifics of these benefits, such as the percentage of the covered salary, the organization responsible for the payment, and the duration the compensation, vary widely between nations. In the Netherlands, the country for which we conducted our research, employers are obligated to continue paying at least 70% of the salary of sick employees for a period of 104 weeks (= 2 years). However, due to collective labor agreements, employers generally cover 100% of the worker’s salary in the first year of sickness absence, and then reduce it to 70% in the second year. In the sequel we will refer to this reduction in salary as a *(financial) incentive* to return to work.

In our study on gender differences in sickness absence [4] we observed a peak in the recovery rate around one year of sickness absence, thereby corroborating the timing of the salary reduction. (We define recovery rate as the fraction of employees that recover in a certain period. In survival analysis this is also known as the hazard rate [5]). This observed correlation is in agreement with psychosocial and economic theories that model work and salary in terms of a balance between the demands and the rewards of work ([6–8]). In other words, our data indicates that the financial incentive instigates a process in which employees reassess the weight they attribute to work on the one hand and salary on the other.

In the current paper we use the data of approximately six million absence cases to make a mathematical model that estimates employee sensitivity to this incentive, thereby providing quantitative support for the just mentioned theoretical balance models. Next, we use our model to simulate the effect of incentives implemented at various *times* and for various levels of *sensitivity* to the incentive. The underlying idea is that if sick employees reassess the balance in the intial stages of sickness absence, not just the employees benefit but also the employers and society as a whole. Finally, we assess the overall influence of the incentivy on two key outcome measures: average sickness absence duration and associated cost. Our approach can be generalized to other countries and/or be used to compare the quantitative impact of other types of incentives and illness policies.

As said, our work relates to psychosocial models that can be used to understand the effects of imbalances in the workplace. Notable works in this area include the demand-control model [8], the conservation of resources model [9], and the effort-reward imbalance model [6, 10]. These models all propose that a certain imbalance can lead to stress and associated health conditions. In particular, the effort-reward imbalance model offers insight into the consequences of salary reductions, as this model focuses on the reciprocity of transactions between employer and employee. The anticipated decrease in reward (namely, the announced salary reduction) appears to shift the balance towards recovery.

Besides psychosocial models, economic models have been developed to study the role of salary and financial incentives in determining work attendance. Common economic models that adopt this perspective include the labor supply model and the theory of moral hazard [11–13]. In the labor supply model, an employee decides whether to work or not by comparing the utility of leisure and consumption on one hand to the utility of labor income on the other. In the case of sickness, this decision-making process might be influenced by various factors, such as the current income, sickness benefits, health status, and working conditions [11, 12]. Economically, sickness absence can also be understood through the theory of moral hazard, where employees might exploit sickness benefits without being genuinely ill. If alterations in sickness benefits or monitoring policies lead to a change in workers’ absence behavior, this could be indicative of moral hazard being present. Such a situation may necessitate careful consideration of the sickness absence policies to align the interests of both employer and employee, ensuring that genuine illness is appropriately supported without opening opportunities for exploitation [13].

In other European countries similar relationships between sickness absence policies and sickness absence behavior have been observed [13–18]. Henrekson et al. [14] analyzed data from 1955–1999, and observed that more generous sick-pay leads to increased work absences and vice versa. Similarly, Johansson and Palme [13, 15] found in Sweden that higher absence costs to sick-listed employees reduced both sickness absence frequency and duration. Puhani et al. [16] observed a two-day reduction in absence duration when sickpay was reduced from 100% to 80%. Ichino et al. [19] noted that after the end of the probabtion period, which indicates higher job security, absenteeism increased. Conversely, Ziebarth et al. [20] found no signficant effect of financial cuts on long-term sickness absence. These studies demonstrate the impacts of sick pay adjustments in their respective countries, though the applicability of their findings to other countries may be limited.

The paper is organized as follows. In Section 2 we describe the data we use for our study. Section 3 develops the quantitative model to estimate the influence of a reduction in salary on the recovery rate, the average sickness absence duration, and associated cost. In Section 4 we analyze by means of simulation the influence of this incentive as a function of the sensitivity and the week in which this incentive is implemented. Section 5 provides a conclusion and discusses our findings.

## 2 Data

In this section, we first provide an overview of the sickness insurance system in the Netherlands. Subsequently, we describe the data that we are using for our analysis.

### 2.1 Sickness insurance in the Netherlands

In the Netherlands, all employers have to arrange that their employees have access to occupational health care, in most cases provided by an occupational health service (OHS). An OHS takes care of the registration of sickness absence, and the consultation and guidance of sick-listed employees. When an employee is reported sick, this is recorded in the OHS sickness absence register. Sickness absence can result from both work-related and non-work-related physical or mental illnesses or injuries. The majority of observed sickness absence cases are of short-term duration, i.e., less than two weeks. These instances are primarily caused by medical conditions such as upper respiratory infections or gastrointestinal disturbances. When the sickness absence period lasts longer, an occupational health physician (OHP) is consulted for a return to work advice. The OHP records the diagnosis of the sickness absence in the OHS register.

In the Netherlands, employers are financially responsible for the salaries of sick employees for a period of 104 weeks. During these two years, employees receive at least 70% of their last earned wages. Due to collective labor agreements, most employers cover 100% of the worker’s salary in the first year of sickness absence and reduce the salary to 70% in the second year. A minority of companies opt for either an earlier reduction in salary or continue to pay 100% throughout the entire sickness period. After 24 months, the employment contract typically comes to an end, and the employee can apply for a disability pension provided by the Employee Insurance Agency (UWV).

### 2.2 Study population

In this study, we use data from the sickness absence register of Arbo Unie (accessed 17 March 2024), one of the largest occupational health services (OHS) in the Netherlands. Arbo Unie maintains a longitudinal, dynamic database covering approximately one million workers across various occupational sectors throughout the Netherlands. The authors did not have access to information that could identify individual participants at any stage during or after data collection.

Our dataset includes records of sickness periods of employees who reported sick between January 2004 and December 2017. The starting point of 2004 was chosen due to significant changes in Dutch sickness absence policies that occurred that year. The data collection concludes in December 2017, enabling us to include all cases of sickness absence lasting up to 104 weeks prior to the onset of the COVID-19 pandemic.

We follow the guidelines defined by the UWV, such that consecutive absences occurring within a 28-day span are considered a single absence period. For each period of sickness absence, we determine the percentage of part-time work and the duration of the sickness absence.

For our subsequent analysis, we exclude cases diagnosed with pregnancy or pregnancy-related diseases, cases with work contracts of fewer than 4 or more than 48 hours per week, cases involving individuals younger than 18 or older than 65, and cases with sickness periods that have a negative duration or last longer than three years. Furthermore, we truncate sickness periods exceeding 104 weeks to 104 weeks exactly, aligning with employer financial responsibilities in the Netherlands.

## 3 Methods

First we provide an overview of the method in Section 3.1. Sections 3.2 and 3.3 provide the mathematical basis of this approach and express the performance measures in terms of the recovery rate.

### 3.1 Overview

**Fig 1:**
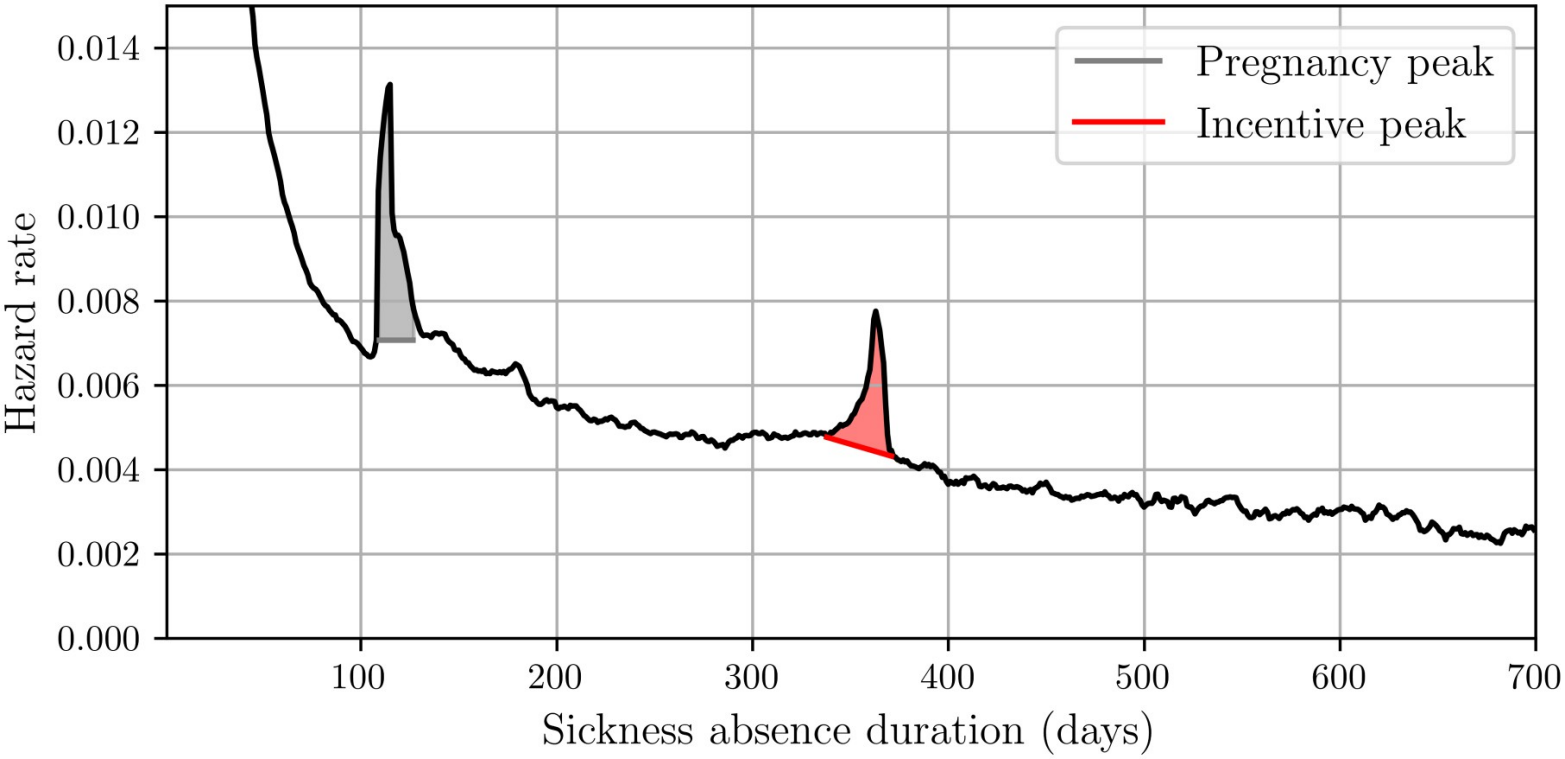
The daily recovery rate *h* as a function of time (in days). The left peak (around 112 days = 16 weeks) corresponds to pregnancy leave, the right peak (around one year) coincides with the installment of the reduction in salary for most employees.

In this section, we develop a model to estimate the effect of financial incentives on average absence duration and associated costs. For this, we first obtain the recovery rate from the measured sickness durations from the sickness absence register, and we plot this rate in Fig 1. In this graph we see two clear peaks. The first peak corresponds to pregnancy leave, which lasts for 16 weeks in the Netherlands. (Although we excluded cases that are diagnosed with pregnancy, many pregancy cases are not seen by the OHP and are therefore still included in our dataset). We attribute the second peak to a financial incentive, as the majority of employees see their salary reducted after exactly one year of sickness absence.

As a second step, we draw a straight line between the start and the end of the peak around one year. This line serves as a baseline to which we compare the measured recovery rate: we define the effect (*e*_*i*_) of the incentive on day *i* as the ratio between the recovery rate measured at day *i* and the baseline at day *i*. Once we have determined the effect *e*_*i*_ associated with the peak in Fig 1, we can explore hypothetical situations by applying the effect at different times. Thirdly, we introduce a sensitivity factor *η* that captures how strongly employees respond to the financial incentive. When *η* = 1, we obtain the measured effect of Fig 1, but when *η* > 1 (*η* < 1) the respons is stronger (weaker).

### 3.2 Estimating the size of the incentive

We first calculate the daily recovery rates based on our sickness absence data. From this data, we determine the number *R*_*t*_ of employees still absent from work due to sickness after *t* days for day *t* = 1, 2, 3, …728 (104 weeks). The recovery rate for day *t* then becomes *r*_*t*_ = (*R*_*t*−1_ − *R*_*t*_)/*R*_*t*−1_, since *R*_*t*−1_ is the number of employees who could potentially return to work on day *t*, and *R*_*t*_ represents the number of employees who remain absent due to sickness after day *t*. (*R*_0_ is the number of employees who have registered as sick; thus *R*_0_ forms the initial population of employees absent due to sickness).

As it turns out, the days on which employees report recovery are not evenly distributed across weekdays. There is a higher percentage on Mondays, resulting in recovery rates that display a weekly pattern with spikes on days 7, 14, 21, and so on. To smooth this out, we apply a one-week moving average filter. Hence, in the sequel, we use the adjusted recovery rate

$$\begin{array}{c} h_{t}=\frac{1}{7}\sum_{i=-3}^{3}r_{t+i},\;\text{for}\;t=4,5,\ldots ,725. \end{array}$$ (1)

As we cannot apply the weekly smoothing filter at the start and end of the period we set *h*_*t*_ = *r*_*t*_ for *t* = 1, 2, 3 and *t* = 726, 727, 728. For notational ease, we write *h* to refer to the adjusted recovery rates of all days. Fig 1 displays the graph of *h*.

Once we have the smoothed recovery rate *h*, we use it to estimate the extent to which salary reduction affects the recovery rate. In Fig 1 we observe that the recovery rate increases from approximately 4 weeks before the peak at day *p* = 365 until one week after the peak. This suggests that the incentive influences the recovery rate for a total of 5 weeks. To quantify the magnitude of the peak, we make a baseline recovery rate by drawing a straight line from the recovery rate *h*_*p*−28_, i.e., 4 weeks before the peak, to *h*_*p*+7_. Specifically, for day *i* within the timeframe where the incentive affects the recovery rate, we define the baseline recovery rate $\tilde{h}$ as

$$\begin{array}{c} {\tilde{h}}_{i}=h_{p-28}+\frac{h_{p+7}-h_{p-28}}{35}\left(i-\left(p-27\right)\right). \end{array}$$

In words, we take ${\tilde{h}}_{i}$ as the recovery rate at day *i*, assuming no incentive was present at day *p*.

Using the calculated baseline, we define the effect of an incentive implemented on day *p* = 365 on the recovery rate at day *i* as follows:

$$\begin{array}{c} e_{i}=\{\begin{array}{cc} h_{i}/{\tilde{h}}_{i}, & \text{for}\;i=p-27,p-26,\ldots ,p+7,\\ 1, & \text{elsewhere.} \end{array} \end{array}$$ (2)

This means that for the specified days around day *p* (from *p* − 27 to *p*+7), the incentive modifies the recovery rate by a factor *e*_*i*_, as we compare the observed recovery rate *h*_*i*_ to the baseline recovery rate $\tilde{h_{i}}$. On all other days, *e*_*i*_ is set to 1, indicating no effect.

### 3.3 Sensitivity and timing of the effect of the incentive

In Section 4.1 below, we provide motivation for the idea that the influence of the incentive can be stronger when applied earlier in the absence period. To incorporate this phenomenon, we introduce a sensitivity factor *η*: when *η* > 1, employees respond stronger to the incentive than as reported in Fig 1, whereas if *η* < 1, the sensitivity is smaller. When *η* = 0, a financial incentive has no effect at all. By multiplying the effect in Eq (2) by *η*, we can easily include the sensitivity.

We next model the effect of applying the financial incentive on an arbitrary day *k* during the first year of sickness absence. Noting that the effect begins four weeks before the peak and ends one week after the peak, we require that 28 ≤ *k* ≤ 365. To apply the incentive on day *k*, we simply shift the effect *e* on the recovery rate accordingly by setting the recovery on any given day *j* as

$$\begin{array}{c} h_{j}\left(k,\eta \right)=\eta e_{j-k+365}h_{j},\;\text{for}\;j=k-27,k-26,\ldots ,k+7. \end{array}$$ (3)

That is, we initially observe *h*_*j*_, but with the assumed implementation of the incentive on day *k* and sensitivity *η*, the modified recovery rate becomes a factor *ηe*_*j*−*k*+*p*_ larger.

### 3.4 Performance indicators

From a (modified) recovery rate *h* we can compute three performance measures of interest. The first is the survival function

$$\begin{array}{c} S_{t}\left(k,\eta \right)=\prod_{i=0}^{t}\left(1-h_{i}\left(k,\eta \right)\right)\;\text{for}\;t=0,1,2,\ldots ,\text{728}, \end{array}$$ (4)

where we set *h*_0_ = 0 to initialize the definition. Second, the average absence duration follows directly from the survival function:

$$\begin{array}{c} \mu \left(k,\eta \right)=\sum_{t=0}^{t=728}S_{t}\left(k,\eta \right). \end{array}$$ (5)

Third, if there is a cost *c*_*t*_ associated to *S*_*t*_ employees still being absent on day *t*, the total cost is

$$\begin{array}{c} C\left(k,\eta \right)=\sum_{t=0}^{t=728}c_{t}S_{t}\left(k,\eta \right). \end{array}$$ (6)

It remains to estimate the daily cost *c*_*t*_. For the purpose of our paper, we assume that the incentive manifests as a salary reduction to 70% of the usual salary, in alignment with the policies of many Dutch companies. Furthermore, we take 26 euros per hour for the daily salary calculation, as this is the average Dutch hourly income between 2004 and 2018 [21]. Finally, we adjust for part-time contracts by multiplying by 82%, as this is the average part-time percentage of the employees in our dataset. Consequently, the cost prior to the implementation of the incentive is *c*_*t*_ = 0.82 × 8 × 26 euros per working day, while after the implementation of the incentive at time *t*, the cost is reduced to 70% of that.

It is worth noting that we do not include the incentive’s impact on other related expenses, such as production loss, costs of hiring replacements, or costs associated with the occupational health service.

### 3.5 Ethical approval

The Institutional Review Board of the Faculty of Economics and Business at the University of Groningen concluded that ethical clearance was not necessary for this study because the Medical Research involving Human Subjects Act does not apply to studies of anonymised register data. Therefore informed consent to participate and consent for publication were not applicable. Consequently, the Board has provided a written statement of no objection.

## 4 Results

In this section, we present the results of how the timing and sensitivity of the aforementioned financial incentive affect the average absence duration and cost. For the timing, we vary the day (the *k* in Eq (3)) at which the incentive is applied, from day 28 to 365 after reporting sick. To analyze the sensitivity for the incentive, we consider four scenarios. The first scenario assumes the sensitivity for the incentive is zero, that is, the financial incentive does not influence the recovery rate at all. We use this scenario solely as a reference. The second scenario uses the default sensitivity *η* = 1, for which we use the effects of the incentive on the recovery rate as measured by the method of Section 3.2. In the third and fourth scenarios, the default sensitivity is increased by 25% and 50%, respectively, i.e., *η* = 1.25 and *η* = 1.5.

The rationale behind these last two scenarios is that the sensitivity to a financial incentive is likely larger than the measured sensitivity, for at least two reasons. First, in the Netherlands, only about 70% of all companies choose to lower the salary after exactly one year. Some companies opt to reduce the salary earlier than one year, while others maintain the normal salary throughout the entire sickness period [22]. Therefore, if all companies, instead of just 70%, applied the salary reduction after exactly one year, we would expect the peak around one year to be higher.

Second, the group of employees who have not recovered after a year of sickness absence includes those with severe (mental and/or physical) health conditions. This group is presumably less sensitive to financial incentives, which is supported by the findings in [20].

**Fig 2:**
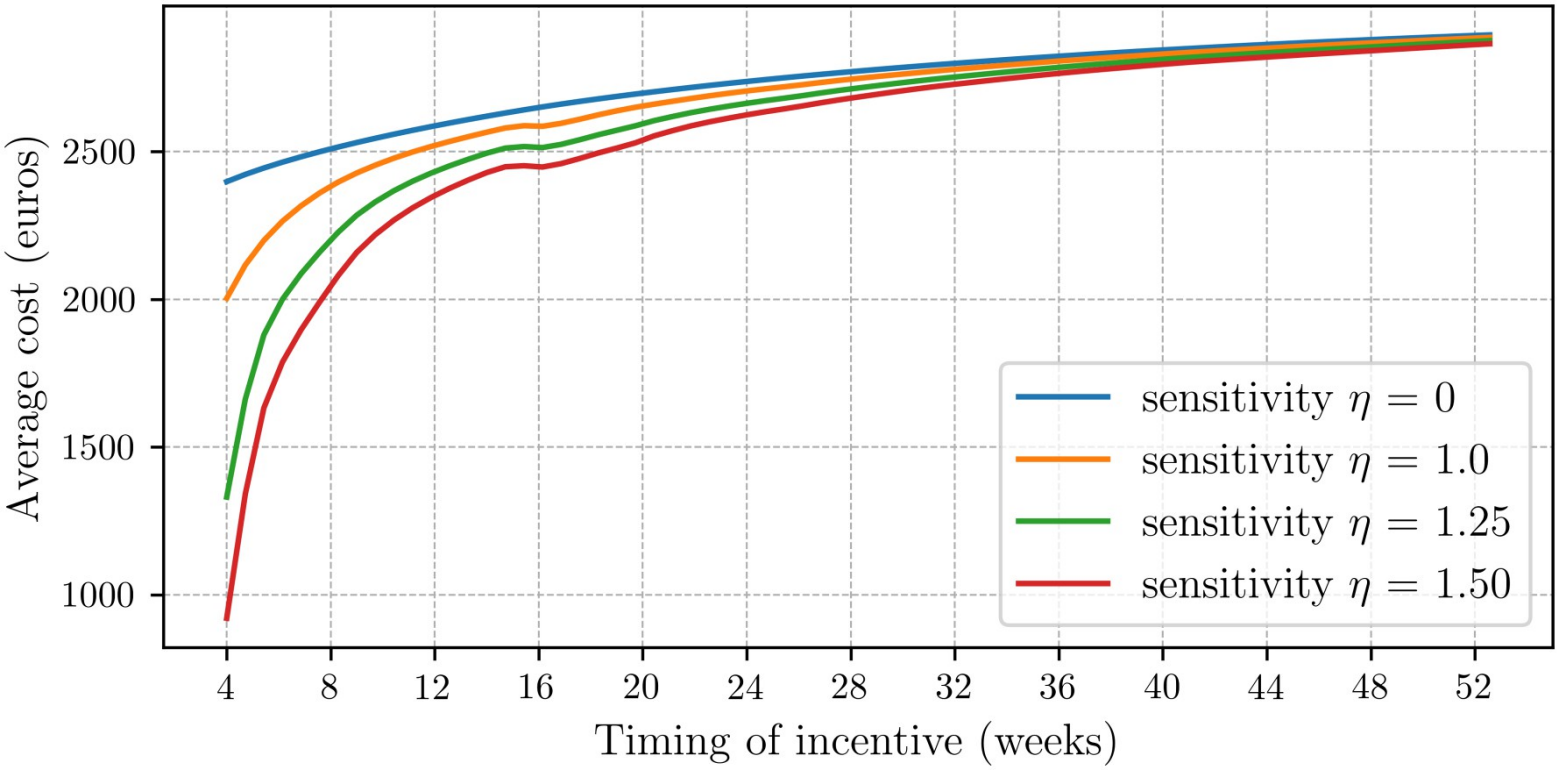
Effect of incentive on average absence duration. These graphs show the average absence duration as a function of the timing of the incentive across all four scenarios. Applying incentives early in the sickness period considerably affects the average absence duration, whereas incentives implemented later have only a minimal effect.

**Fig 3:**
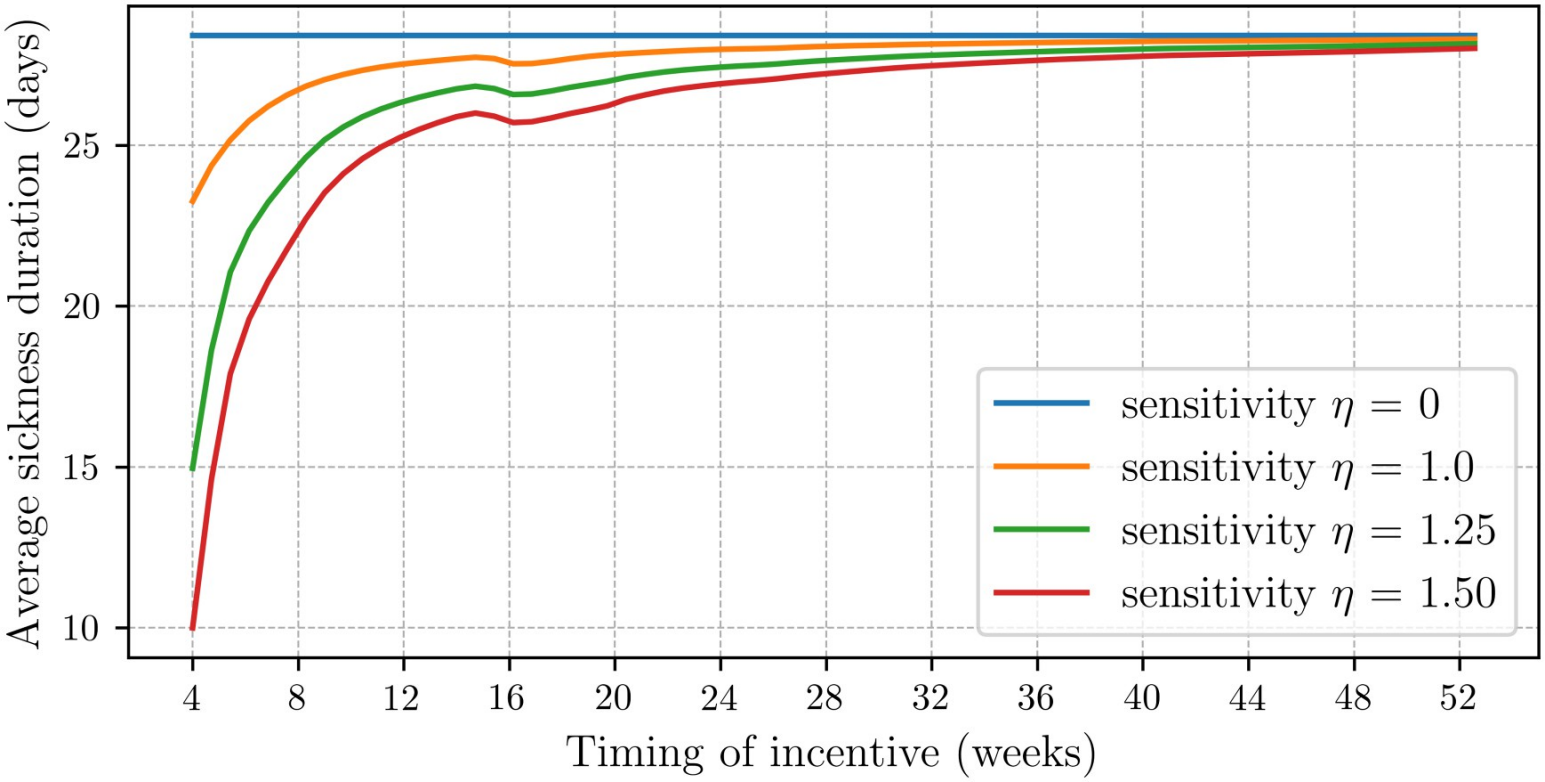
Effect of incentive on average cost. The average cost per absence period as a function of the timing the incentive is applied for all four scenarios. Clearly, the average cost decreases considerably when incentives are implemented earlier than after one year.

Section 4.1 presents the descriptive statistics. Section 4.2 discusses how, across the four scenarios, the average sickness duration varies as function of the timing of the incentive. Section 4.3 cost focuses on the average cost. The main results are contained in Figs 2 and 3 which show the effects of incentives on the absence duration and costs. Note that the graphs show a small dip around 16 weeks which, as said, can be directly attributed to the pregnancy leave peak, cf, Fig 1.

### 4.1 Descriptive statistics

Between January 2004 and December 2017, there were 6,135,891 sickness absence periods, of which 5,628,325 periods met our inclusion criteria (aged between 18 and 65, working hours between 4 and 48 per week, a sickness duration between 1 day and 104 weeks, excluding cases diagnosed with pregnancy or pregnancy-related diseases). Of these cases, 55% are male, and 45% are female. The mean absence duration is 28 days, and the median absence duration is 5 days. The proportion of sick employees who have recovered at specific points in time is as follows: 83% after four weeks, 93% after three months, 96% after six months, and 99% after one year. This indicates that only a very small percentage remains unrecovered after one year of sickness absence.

### 4.2 Effect on absence duration

Fig 2, for the four different sensitivity scenarios, the average sickness duration as a function of the day the financial incentive is applied. Clearly, in the first scenario, the average absence duration remains constant at 28.4 days, because in this scenario the sensitivity *η* = 0.

In the other scenarios, the average sickness duration steadily increases (except for a dip around 16 weeks due to pregnancy leave). All four graphs converge after one year, indicating that applying the incentive after one year has a minimal effect. This is because the number of employees still sick after one year is very small (1.4%). As the majority of sickness absences are of short duration, these absences are unaffected by any incentive introduced at later stages. Thus, if the incentive aims to reduce the average absence duration, it should be implemented much earlier, thereby affecting a significantly larger population.

When the incentive is applied after approximately six months, the absence duration is only slightly reduced to 28.0 days in the second scenario, and to 27.5 and 27.0 days in the third and fourth scenarios, respectively, cf. Table 1. However, when the incentive is applied after four weeks, by which time approximately 17% of the employees have not yet recovered, we observe a substantial reduction in the number of absence days. In the second scenario, the absence duration decreases to 23.2 days, and further to 14.9 and 10.0 days in the third and fourth scenarios, respectively. Although our model does not allow for evaluating the effects of applying the incentive earlier than four weeks, we expect a very significant impact if incentives are applied immediately at the start of the sickness period.

**Table 1:** Incentive effects. Simulation results for different scenarios, where each scenario assumes a different sensitivity to the incentive. The first column gives the timing the incentive is implemented. The other columns show the average absence duration (in days) and the average cost per absence period (in euros) for each scenario.

sensitivity	*η* = 0		*η* = 1.0		*η* = 1.25		*η* = 1.5	
timing	duration	cost	duration	cost	duration	cost	duration	cost
4 weeks	28.4	2398	23.2	2003	14.9	1330	10.0	920
3 months	28.4	2604	27.6	2544	26.5	2465	25.6	2393
6 months	28.4	2754	28.0	2725	27.5	2687	27.0	2652
1 year	28.4	2893	28.2	2885	28.1	2873	27.9	2863
2 years	28.4	2990	28.3	2989	28.3	2989	28.3	2989

As a rule of thumb, to achieve a reduction of around 10% in average absence duration, the incentive should be applied between 1 and 3 months, depending on the sensitivity.

### 4.3 Effects on cost per absence period

Fig 3 shows the effect of an incentive on the average cost per absence period across our four scenarios. The upper graph in Fig 3 presents the average cost under the initial scenario, where employees are entirely insensitive to the incentive. Interestingly, despite the incentive having no effect on the absence duration, cf. Fig 2, it does influence the average cost. The average difference in cost per absence period between implementing the incentive after three months versus one year is about 10% (≈ (2893 − 2604)/2893). Of course, this reduction is caused by the decrease in salary paid when the incentive is implemented earlier. For the same reasons, the cost per absence period increases to 2990 euros when the incentive is not applied at all.

Fig 3 demonstrates that the effect on average cost is substantial under all scenarios. In the second scenario, with assumed default sensitivity to the incentive, the average cost decreases from 2885 to 2725 euros when the incentive is introduced after six months, marking a 6% reduction in cost per sickness absence period. It is important to note that the cost is an average over all absence periods, including short ones where the cost remains unchanged. The average cost per absence period decreases further to 2003 euros when the incentive is introduced after four weeks.

In scenarios with increased sensitivity, i.e., *η* = 1.25 and *η* = 1.5, the effects are even more pronounced. In the third scenario, we estimate that the cost per sickness period decreases to 2687 euros when the incentive is applied after six months, and further to 1330 euros when it is applied after four weeks, cf. Table 1. In the fourth scenario, the average cost per sickness period decreases to 920 euros when the incentive is applied after four weeks, indicating a cost reduction of almost 70%. Given the high percentage of employees currently absent from work, this decrease in the average cost per absence period can significantly impact the total expenses incurred from sickness absence.

## 5 Conclusions & discussion

In this paper, we estimate the effect of financial incentives on sickness absence duration and associated cost. For this, we develop a model to quantify the effect of such incentives on the recovery rate, and from the latter, we derive the average absence duration and associated cost.

Our findings show that for incentives to effectively reduce the duration of absence, they must be implemented early in the absence period. Incentives implemented after three month can potentially reduce the average sickness duration by approximately 2 days, which is a reduction of about 7%. Incentives implemented later in the absence period have little impact on the average absence duration. Although the effect on the average absence duration is relatively small, the impact on average cost is more substantial, even when applied at later moments during absence. For example, implementing the incentive after six months of sickness absence leads to an average cost reduction of 6% per sickness absence period. Considering the high proportion of employees currently on sick leave, a decrease in average cost per absence period can significantly reduce the total expenditure on sickness absence.

The results from our study align with the general view that sickness policies impact the duration of sickness absence [13–18]. Most of these studies estimate the effects of incentives on both absence duration and frequency. Ziebarth et al. [17] find a significant reduction in absence frequency, but only a minor reduction in average absence duration. In another study, they report that financial incentives mainly affect employees with short-term absences, and that their influence on long-term sickness absence is not significant [20]. This is consistent with our findings that incentives should be applied early in the sickness period to have a considerable effect on absence duration. We expect an even larger effect when incentives are applied immediately, which will also impact absence frequency.

In addition to analyzing the impact of financial policies on absence duration and associated cost, it is important to assess the broader implications of implementing new policies on the health and well-being of both individual employees and the workforce as a whole. The potential health consequences of financial incentives on employees can be understood by using the effort-reward imbalance model [6, 10]. This model states that health issues may emerge from an imbalance between the effort an employee invests in their work and the rewards they receive in return. Furthermore, overcommitment is a key factor in this framework, significantly influencing the effort-reward balance. Our findings indicate that a stated reduction in rewards leads to a higher recovery rate, suggesting an improvement in health conditions sufficient to facilitate recovery. This apparently paradoxical effect might be explained by measures taken to address the imbalance that had developed. Such adjustments may involve a reevaluation of rewards—where the announced decrease motivates employees to appreciate their existing salary more–—a change in commitment, or a modification in future efforts, thereby correcting the imbalance.

From the viewpoint of both colleagues and the organization, implementing financial incentives early may offer several benefits. To begin with, such a strategy can shorten the duration of sickness absences, thereby alleviating the load on the rest of the workforce. This approach will prevent the ‘domino effect,’ a scenario where the sickness absence of one employee leads to an increased workload for the other employees, creating imbalances and possibly resulting in additional absences.

Moreover, the saved financial resources could be strategically used to enhance the well-being of all employees. This enhancement can be achieved either by reducing indiviual workloads (through hiring additional personnel or investing in new machinery) or by providing additional rewards (by increasing wages, or making investments in employee satisfaction, including preventive health initiatives).

However, in addition to these advantages, advancing the financial incentive could have unfavorable consequences. The anticipated reduction in salary might pressure employees to return to work while still experiencing sickness symptoms. This could negatively affect employee well-being and result in recurrent, short-term sickness absences. Furthermore, it could lead to presenteeism, where employees are physically present at work but have reduced productivity.

This study has some limitations that provide directions for further research. First, it does not account for a range of factors that could influence recovery rates and the sensitivity to financial incentives. These include employee demographics such as gender, age, diagnosis, socio-economic status, and overall health status, alongside work-related characteristics like job nature, company size, and types of employment contracts. Future research could benefit from examining these variables to see whether such incentives disproportionately affect certain subgroups.

Second, our analysis lacks detailed information on salaries, specifically regarding the magnitude and timing of salary reductions. While we anticipate a more significant effect if all companies were to implement the incentive around one year, having detailed salary information could enhance the precision of our findings.

Third, as this research is partly based on a simulation model, it relies on assumptions about the temporal sensitivity to incentives. Although we motivate that the effective sensitivity is likely greater early in the absence period than the observed sensitivity (see Section 4.1), empirical validation is needed to substantiate these claims.

Finally, our study does not examine how financial incentives compare with other sickness absence policies that might be equally effective. In particular, strategies addressing the effort-reward imbalance could prove beneficial. These strategies might involve offering more personalized guidance during sickness absence or emphasizing support for (partial) return to work. Future research should aim to compare these different approaches to determine the most effective strategies in terms of health outcomes and cost-efficiency. Achieving this objective requires a collective effort from employees, employers, and labor unions to maintain an open-minded perspective and a shared interest in enhancing health while simultaneously reducing costs.
