# Supplementary material for: Effect of financial incentives on the cost and duration of sickness absence
Source: PLoS One. 2024 Jun 13;19(6):e0305235. doi: 10.1371/journal.pone.0305235 (PMC11175486; doi:10.1371/journal.pone.0305235)
Supplement: Attachment — Submitted filename: response_letter.pdf [file pone.0305235.s001.pdf]

Response to referee on ‘Effect of financial incentives on the cost and duration of sickness absence’

Sheila Timp, Nicky van Foreest, Willen van Rhenen

April 3, 2024

We thank the referee for the valuable suggestions, which have led to extensive revisions in the introduction, results, and conclusion sections of our manuscript. Given the extent of these modifications, the version that highlights the changes may not be useful. With the modifications, we hope to have addressed all the recommendations and comments of the referee and the editor. For the convenience of the referee, we follow the sections of the referee to structure our answers.

1 Referee: major comments

1.1 Motivation

The referee has some comments on the motivation of the paper. The first question is how The Netherlands compares to other countries. Despite the international variation in sickness absence policies, research from multiple countries demonstrates that financial incentives significantly influence sickness absence rates. Moreover, our method, which examines the effect of incentives on the recovery rate, offers a framework that can also be applied to other countries and/or be used to compare the quantitative impact of other types of incentives and absence policies.

We have clarified this point further in the introduction section (page 2, paragraph 3; page 3, paragraph 1).

Secondly, the referee asks how the paper contributes to and expands upon the exisisting literature. For this we follow the helpful recommendations of the referee; please see below.

1.2 Literature

I recommend incorporating references to well-known literature on job demands, such as the seminal work by Karasek (1979 onwards), and effort-reward imbalance theory as proposed by Siegrist (1996, 2002, 2004), as potential sources influencing sickness absence. In the second part of the introduction we now discuss this line of research to position our paper with respect to the models discussed in this work (page 2, paragraph 4). In the conclusion we come back to these models to provide suggestions for further research (page 10, last paragraph).

1.3 Data and period of analysis

The data provided spans from January 2004 to December 2020, as far as I've gleaned from the paper. However, it's noteworthy that the final year of observations coincides with the

onset of the COVID-19 outbreak. Our data intially included results from years of the COVID pandemic. To ensure that our data are not compromised by pandemic-related issues, we follow the suggestion of the referee and exclude these years from our analysis. Our revised dataset now covers the period from January 2004 to December 2017. We note that this adjustment only marginally affects our findings, aligning with the referee's expectations.

The referee asks for the definition of the recovery rate. We follow the standard definition of recovery rate as used in survival analysis, actuarial sciences, manufacturing and reliability analysis, etc. Our definition of recovery rate is the same as the commonly used words 'hazard rate' and 'survival rate'. We added this information in the text (page 2, second paragraph) and provide a reference ([Collett(2015)]).

1.4 Results

The results slightly changed as the dataset now covers a different period (excluding the COVID-19 years). These changes however did not affect our findings. We revised the results section; we more thoroughly interpret our findings with the help of the figures an added Table 1 to provide more numerical details about our findings.

Alternatively, enriching and link the final discussion section with a more thorough analysis and interpretation of the results could also elevate the overall depth and richness of the paper. In the discussion section, we analyze the results and interpret them using the effort-reward imbalance model. This approach has enriched the paper, and expanded the scope of our findings.

1.5 Language

We apologize for the errors in the writing. We hope that the resubmisison has much improved in this regard.

2 Referee: minor comments

We dealt with all minor comments. We italize the comment of the referee, and add our responses in normal font.

1. *Is there a rationale behind citing some references directly in the paper while assigning numerical references to others, which are then listed at the end? I notice that this is handled differently in the first paragraphs of the paper compared to the rest.* We made this consistent throughout the paper

2. *Last paragraph, pag.1: I would change "In literature" with "In the literature" .* Done

3. *Last paragraph pag, 2: I think that the description of how sickness leave works in the Netherlands should be eliminated from here since it is already explained in depth in the paragraph named"" at pag, 3. So I suggest to drop this sentence "In The Netherlands, the employer is financially responsible for the salary of a sick employee during a period of 104 weeks. Within these 104 weeks, employees are guaranteed at least 70% of their last earned salary. Due to collective labor agreements, employers in general cover 100% of the worker's salary in the first year of sickness", from pag, 2.* Done.

4. *Pag. 6 Scenarios. What scenarios are intended by the authors? Please suggest an alternative title.* We removed this title

5. *Pag. 6 Descriptive statistics? ..instead of Results?* Done

6. *Pag.8" . . . in another study they find that a financial incentive mainly affected employees with short term.." only two studies are cited to support your results. Expand on this could help to motivate the paper.* Done.

7. *Pag. 9 change this "..the perspective of colleagues and the organization as a whole, advancing the introduction of an incentive can yield certain benefits, when the resulting savings are wisely allocated" in "From both the perspective of colleagues and the organization as a whole, introducing an incentive scheme can yield several benefits, particularly when the resulting savings are allocated wisely."* Done.

3 Response to the editor

3.1 Main comment

In particular, after reading the paper I agree with the suggestions of the referee that the the paper needs substantial additional effort to improve its quality, in terms of related literature, conceptual framework, methodology and interpretation of results.. We thoroughly revised the manuscript using the comments from the referee to enhance its quality.

3.2 Requirements

1. *PLOS ONE style requirements.* We use the LaTeX style file to ensure the manuscript meets PLOS ONE's style requirements

2. *Follow PLOS ONE guidelines on code sharing* NA

3. *Please update your submission to use the PLOS LaTeX template.* Done

4. *PLOS' Data Policy states that authors cannot be the sole named individuals responsible for ensuring data access* We added the emailaddress of the department that is responsible for the data access.

5. *Please include your full ethics statement in the 'Methods' section of your manuscript file.* We added the full ethics statement, and mentioned that we obtained written consent.

6. *Please upload a copy of Figures 1 to 3, to which you refer in your text on pages 4 and 7.* Done

7. *Please include captions for your Supporting Information files at the end of your manuscript* All figures/tables are included in the main text and not added as supporting information.

References

[Collett(2015)] David Collett. *Modelling Survival Data in Medical Research.* Taylor & Francis Group, LLC, Bristol, UK, 2015. ISBN 978-1-4987-3169-0.
