# Supplementary material for: Effect of financial incentives on the cost and duration of sickness absence
Source: PLoS One. 2024 Jun 13;19(6):e0305235. doi: 10.1371/journal.pone.0305235 (PMC11175486; doi:10.1371/journal.pone.0305235)
Supplement: Attachment — Submitted filename: response_letter.pdf [file pone.0305235.s002.pdf]

Response to reviewer on 'Effect of financial incentives on the cost and duration of sickness absence'

Sheila Timp, Nicky van Foreest, Willen van Rhenen

May 17, 2024

We thank the reviewer for the textual corrections, all of which we have incorporated. For the convenience of the referee, below we italicize the comments from the reviewer and add our responses in normal font.

1. *Pag.1, first paragraph. I think that this phrase is not clear: "An important component of these policies is related to the financial compensation and the duration and height for which it is provided to absent employees", probably the authors mean:" A key aspect of these policies concerns the financial compensation provided to absent employees, including its amount and the length of time it is offered.* Done.

2. *Page 9 last paragraph, change into or improve clarity: Our findings indicate that a stated reduction in rewards leads to a higher recovery rate, suggesting an improvement in health conditions that sufficiently facilitates recovery.* Done.

3. *Pag 10, fifth paragraph: change salaries into wages.* Done.
