# Supplementary material for: Effect of financial incentives on the cost and duration of sickness absence
Source: PLoS One. 2024 Jun 13;19(6):e0305235. doi: 10.1371/journal.pone.0305235 (PMC11175486; doi:10.1371/journal.pone.0305235)
Supplement: Peer review history

# Peer review: Decision Letter 0

**DOI:** 10.1371/journal.pone.0305235.r001

**Authors:** Paolo Ghinetti

6 Mar 2024

PONE-D-23-38392Effect of financial incentives on the cost and duration of sickness absence.PLOS ONE

Dear Dr. Timp,

Thank you for submitting your manuscript to PLOS ONE. After careful consideration, we feel that it has merit but does not fully meet PLOS ONE’s publication criteria as it currently stands. Therefore, we invite you to submit a revised version of the manuscript that addresses the points raised during the review process.

 Please submit your revised manuscript by Apr 20 2024 11:59PM. If you will need more time than this to complete your revisions, please reply to this message or contact the journal office at plosone@plos.org. When you're ready to submit your revision, log on to https://www.editorialmanager.com/pone/ and select the 'Submissions Needing Revision' folder to locate your manuscript file.

Please include the following items when submitting your revised manuscript:

- A rebuttal letter that responds to each point raised by the academic editor and reviewer(s). You should upload this letter as a separate file labeled 'Response to Reviewers'.
- A marked-up copy of your manuscript that highlights changes made to the original version. You should upload this as a separate file labeled 'Revised Manuscript with Track Changes'.
- An unmarked version of your revised paper without tracked changes. You should upload this as a separate file labeled 'Manuscript'.

If you would like to make changes to your financial disclosure, please include your updated statement in your cover letter. Guidelines for resubmitting your figure files are available below the reviewer comments at the end of this letter.

If applicable, we recommend that you deposit your laboratory protocols in protocols.io to enhance the reproducibility of your results. Protocols.io assigns your protocol its own identifier (DOI) so that it can be cited independently in the future. For instructions see: https://journals.plos.org/plosone/s/submission-guidelines#loc-laboratory-protocols. Additionally, PLOS ONE offers an option for publishing peer-reviewed Lab Protocol articles, which describe protocols hosted on protocols.io. Read more information on sharing protocols at https://plos.org/protocols?utm_medium=editorial-email&utm_source=authorletters&utm_campaign=protocols.

We look forward to receiving your revised manuscript.

Kind regards,

Paolo Ghinetti, PhD

Academic Editor

PLOS ONE

Journal Requirements:

When submitting your revision, we need you to address these additional requirements.

1. Please ensure that your manuscript meets PLOS ONE's style requirements, including those for file naming. The PLOS ONE style templates can be found at 

https://journals.plos.org/plosone/s/file?id=wjVg/PLOSOne_formatting_sample_main_body.pdf and 

https://journals.plos.org/plosone/s/file?id=ba62/PLOSOne_formatting_sample_title_authors_affiliations.pdf

2. Please note that PLOS ONE has specific guidelines on code sharing for submissions in which author-generated code underpins the findings in the manuscript. In these cases, all author-generated code must be made available without restrictions upon publication of the work. Please review our guidelines at https://journals.plos.org/plosone/s/materials-and-software-sharing#loc-sharing-code and ensure that your code is shared in a way that follows best practice and facilitates reproducibility and reuse.

3. Please update your submission to use the PLOS LaTeX template. The template and more information on our requirements for LaTeX submissions can be found at http://journals.plos.org/plosone/s/latex.

4. In this instance it seems there may be acceptable restrictions in place that prevent the public sharing of your minimal data. However, in line with our goal of ensuring long-term data availability to all interested researchers, PLOS’ Data Policy states that authors cannot be the sole named individuals responsible for ensuring data access (http://journals.plos.org/plosone/s/data-availability#loc-acceptable-data-sharing-methods).

Data requests to a non-author institutional point of contact, such as a data access or ethics committee, helps guarantee long term stability and availability of data. Providing interested researchers with a durable point of contact ensures data will be accessible even if an author changes email addresses, institutions, or becomes unavailable to answer requests.

Before we proceed with your manuscript, please also provide non-author contact information (phone/email/hyperlink) for a data access committee, ethics committee, or other institutional body to which data requests may be sent. If no institutional body is available to respond to requests for your minimal data, please consider if there any institutional representatives who did not collaborate in the study, and are not listed as authors on the manuscript, who would be able to hold the data and respond to external requests for data access? If so, please provide their contact information (i.e., email address). Please also provide details on how you will ensure persistent or long-term data storage and availability.

5. Please include your full ethics statement in the ‘Methods’ section of your manuscript file. In your statement, please include the full name of the IRB or ethics committee who approved or waived your study, as well as whether or not you obtained informed written or verbal consent. If consent was waived for your study, please include this information in your statement as well. 

6. Please upload a copy of Figures 1 to 3, to which you refer in your text on pages 4 and 7. If the figure is no longer to be included as part of the submission please remove all reference to it within the text.

7. Please include captions for your Supporting Information files at the end of your manuscript, and update any in-text citations to match accordingly. Please see our Supporting Information guidelines for more information: http://journals.plos.org/plosone/s/supporting-information. 

**Additional Editor Comments:**

In particular, after reading the paper I agree with the suggestions of the referee that the the paper needs substantial additional effort to improve its quality, in terms of related literature, conceptual framework, methodology and interpretation of results.

In the revised version, I ask the author(s) to follow the suggestions of the referee and to address all the points raised in their comments.

[Note: HTML markup is below. Please do not edit.]

Reviewers' comments:

Reviewer's Responses to Questions

**Comments to the Author**

1. Is the manuscript technically sound, and do the data support the conclusions?

The manuscript must describe a technically sound piece of scientific research with data that supports the conclusions. Experiments must have been conducted rigorously, with appropriate controls, replication, and sample sizes. The conclusions must be drawn appropriately based on the data presented. 

Reviewer #1: Partly

**********

2. Has the statistical analysis been performed appropriately and rigorously? 

Reviewer #1: Yes

**********

3. Have the authors made all data underlying the findings in their manuscript fully available?

The PLOS Data policy requires authors to make all data underlying the findings described in their manuscript fully available without restriction, with rare exception (please refer to the Data Availability Statement in the manuscript PDF file). The data should be provided as part of the manuscript or its supporting information, or deposited to a public repository. For example, in addition to summary statistics, the data points behind means, medians and variance measures should be available. If there are restrictions on publicly sharing data—e.g. participant privacy or use of data from a third party—those must be specified.

Reviewer #1: Yes

**********

4. Is the manuscript presented in an intelligible fashion and written in standard English?

PLOS ONE does not copyedit accepted manuscripts, so the language in submitted articles must be clear, correct, and unambiguous. Any typographical or grammatical errors should be corrected at revision, so please note any specific errors here.

Reviewer #1: No

**********

5. Review Comments to the Author

Please use the space provided to explain your answers to the questions above. You may also include additional comments for the author, including concerns about dual publication, research ethics, or publication ethics. (Please upload your review as an attachment if it exceeds 20,000 characters)

Reviewer #1: *************************************************************************************************

****Referee report for PONE-D-23-38392: Effect of financial incentives on the cost and duration of sickness absence.

**************************************************************************************************

Sickness absence has been a steadily growing concern in the public health sphere. Not only does it impact individual lives, but it also bears significant costs for businesses and society as a whole. In this article the authors study how current financial sickness absence policies might be influencing the cost and duration of sickness absence. Additionally, they analyze the implications of different financial policies on these outcomes. Our findings provide valuable insights for healthcare professionals and employers in formulating effective sickness absence policies.

Below are listed more detailed comments, and points that I believe need to be clarified:

**************

Major comments

**************

*Motivation: I have a couple of comments on the motivation of the paper. First, how does the Netherlands compare to the other countries in terms of sickness absence mentioned in the Introduction of the paper? This might be helpful to further motivate the analysis. With respect to motivation also the authors should insist on the originality of the data they use for their analysis. Secondly, how does the paper contribute to and expand upon the existing literature?

*Literature: I recommend incorporating references to well-known literature on job demands, such as the seminal work by Karasek (1979 onwards), and effort-reward imbalance theory as proposed by Siegrist (1996, 2002, 2004), as potential sources influencing sickness absence. These established frameworks provide valuable insights into the interplay between workplace factors and employee health outcomes, offering a robust foundation for understanding the dynamics at play in the context of sickness absence. By drawing upon these seminal studies, the discussion on the potential influences of job demands and effort-reward imbalances on sickness absence can be enriched, thereby enhancing the theoretical underpinning of the paper's analysis.

*Data and period of analysis. The data provided spans from January 2004 to December 2020, as far as I've gleaned from the paper. However, it's noteworthy that the final year of observations coincides with the onset of the COVID-19 outbreak. While this impacts only one year of observations, it's surprising that the authors did not address it at all. One might wonder about the implications of sickness absence during this period and how it could potentially influence the outcomes obtained. It's a crucial aspect that warrants discussion within the paper. Considering the unprecedented circumstances brought about by the pandemic, it's plausible that sickness absence patterns might have deviated significantly from the norm, potentially skewing the results. Therefore, it would be prudent to deliberate on the potential effects of this outlier year on the overall analysis. One recommendation could be to exclude the last year of observation from the analysis altogether. This would ensure a more consistent dataset and potentially mitigate any distortions caused by the unique circumstances of the COVID-19 pandemic. Please discuss also the implications on how would that influence the sickness periods lasting up to 104 weeks?

*Methodology. Recovery rate: is it a simple measure but is it used in the literature? How does the paper improve over the literature? Please clarify an add references to existing studies. For instance, in epidemiology, the recovery rate serves as a crucial metric in understanding the effectiveness of treatments and interventions during disease outbreaks (Smith et al., 2020).

*Results. The description of results presented in the manuscript is cursory and lacks sufficient detail. Strengthening the articulation and elaboration of the results would significantly enhance the clarity and comprehensiveness of the findings. Alternatively, enriching and link the final discussion section with a more thorough analysis and interpretation of the results could also elevate the overall depth and richness of the paper.

*Language: The language is not always clear, and sometime incorrect from the grammatical point of view: it seems that it has not been checked before submission. I think that the paper need rewriting and polish. As an example take, pag 3, sixth paragraph they write :”Our dataset includes records of sickness periods of employees who reported sick between January 2004 and December 2020”. That should be corrected with “Our dataset includes records of sickness periods for employees who reported being sick between January 2004 and December 2020.”, or also pag.7, third paragraph I think that “abence “ is absence.

*************

Minor comments

**************

References: Is there a rationale behind citing some references directly in the paper while assigning numerical references to others, which are then listed at the end? I notice that this is handled differently in the first paragraphs of the paper compared to the rest.

Last paragraph, pag.1: I would change “In literature” with “In the literature” .

Last paragraph pag, 2: I think that the description of how sickness leave works in the Netherlands should be eliminated from here since it is already explained in depth in the paragraph named”” at pag, 3. So I suggest to drop this sentence “In The Netherlands, the employer is financially responsible for the salary of a sick employee during a period of 104 weeks. Within these 104 weeks, employees are guaranteed at least 70% of their last earned salary. Due to collective labor agreements, employers in general cover 100% of the worker’s salary in the first year of sickness”, from pag, 2.

Pag. 6 Scenarios. What scenarios are intended by the authors? Please suggest an alternative title.

Pag. 6 Descriptive statistics? ..instead of Results?

Pag.8” …in another study they find that a financial incentive mainly affected employees with short term..” only two studies are cited to support your results. Expand on this could help to motivate the paper.

Pag. 9 change this “..the perspective of colleagues and the organization as a whole, advancing the introduction of an incentive can yield certain benefits, when the resulting savings are wisely allocated” in “From both the perspective of colleagues and the organization as a whole, introducing an incentive scheme can yield several benefits, particularly when the resulting savings are allocated wisely.”

**************

References

************

Karasek, Jr., R. A. 1979. Job demands, job decision latitude, and mental strain: Implications for job redesign. Administrative Science Quarterly, 24(2): 285-308

Siegrist, J. 1996. Adverse health effects of high-effort/low-reward conditions. Journal of Occupational Health Psychology, 1: 27-41.

Siegrist, J. 2002. Effort-reward Imbalance at Work and Health. In Research in occupational stress and well-being: Vol. 2. Historical and current perspectives on stress and health, edited by Perrewe, P. L. and D. C. Ganster. New York: JAI Elsevier, pp. 261–291.

Siegrist, J., Starke, D., Chandola, T., Godin, I., Marmot, M., Niedhammer, I., and P. Richard. 2004. The Measurement of Effort–reward imbalance at work: European Comparisons. Social Science & Medicine, 58(8): 1483-1499.

Smith, L., et al. (2020). Recovery rates in infectious disease outbreaks: A systematic review. Epidemiology & Infection, 148, e123.

**********

6. PLOS authors have the option to publish the peer review history of their article (what does this mean?). If published, this will include your full peer review and any attached files.

If you choose “no”, your identity will remain anonymous but your review may still be made public.

**Do you want your identity to be public for this peer review?** For information about this choice, including consent withdrawal, please see our Privacy Policy.

Reviewer #1: No

**********

[NOTE: If reviewer comments were submitted as an attachment file, they will be attached to this email and accessible via the submission site. Please log into your account, locate the manuscript record, and check for the action link "View Attachments". If this link does not appear, there are no attachment files.]

While revising your submission, please upload your figure files to the Preflight Analysis and Conversion Engine (PACE) digital diagnostic tool, https://pacev2.apexcovantage.com/. PACE helps ensure that figures meet PLOS requirements. To use PACE, you must first register as a user. Registration is free. Then, login and navigate to the UPLOAD tab, where you will find detailed instructions on how to use the tool. If you encounter any issues or have any questions when using PACE, please email PLOS at figures@plos.org. Please note that Supporting Information files do not need this step.

# Author comment: Author response to Decision Letter 0

**DOI:** 10.1371/journal.pone.0305235.r002

3 Apr 2024

Dear reviewer,

We thank you for the valuable suggestions, which have led to extensive revisions in the introduction, results, and conclusion sections of our manuscript. We hope to have addressed all of your recommendations and comments. Please find our response to the recommendations in the attached letter.

Attachment Submitted filename: response_letter.pdf

# Peer review: Decision Letter 1

**DOI:** 10.1371/journal.pone.0305235.r003

**Authors:** Paolo Ghinetti

13 May 2024

PONE-D-23-38392R1Effect of financial incentives on the cost and duration of sickness absence.PLOS ONE

Dear Dr. Timp,

Thank you for submitting your manuscript to PLOS ONE. After careful consideration, we feel that it was substiantially improved since the first submission, but there are still some minor points that need to be fixed, following the suggestions of the referee. Therefore, we invite you to submit a revised version of the manuscript that addresses the points raised during the review process.

Please submit your revised manuscript by Jun 27 2024 11:59PM. If you will need more time than this to complete your revisions, please reply to this message or contact the journal office at plosone@plos.org. When you're ready to submit your revision, log on to https://www.editorialmanager.com/pone/ and select the 'Submissions Needing Revision' folder to locate your manuscript file.

Please include the following items when submitting your revised manuscript:

- A rebuttal letter that responds to each point raised by the academic editor and reviewer(s). You should upload this letter as a separate file labeled 'Response to Reviewers'.
- A marked-up copy of your manuscript that highlights changes made to the original version. You should upload this as a separate file labeled 'Revised Manuscript with Track Changes'.
- An unmarked version of your revised paper without tracked changes. You should upload this as a separate file labeled 'Manuscript'.

If you would like to make changes to your financial disclosure, please include your updated statement in your cover letter. Guidelines for resubmitting your figure files are available below the reviewer comments at the end of this letter.

If applicable, we recommend that you deposit your laboratory protocols in protocols.io to enhance the reproducibility of your results. Protocols.io assigns your protocol its own identifier (DOI) so that it can be cited independently in the future. For instructions see: https://journals.plos.org/plosone/s/submission-guidelines#loc-laboratory-protocols. Additionally, PLOS ONE offers an option for publishing peer-reviewed Lab Protocol articles, which describe protocols hosted on protocols.io. Read more information on sharing protocols at https://plos.org/protocols?utm_medium=editorial-email&utm_source=authorletters&utm_campaign=protocols.

We look forward to receiving your revised manuscript.

Kind regards,

Paolo Ghinetti, PhD

Academic Editor

PLOS ONE

Journal Requirements:

Please review your reference list to ensure that it is complete and correct. If you have cited papers that have been retracted, please include the rationale for doing so in the manuscript text, or remove these references and replace them with relevant current references. Any changes to the reference list should be mentioned in the rebuttal letter that accompanies your revised manuscript. If you need to cite a retracted article, indicate the article’s retracted status in the References list and also include a citation and full reference for the retraction notice.

[Note: HTML markup is below. Please do not edit.]

Reviewers' comments:

Reviewer's Responses to Questions

**Comments to the Author**

1. If the authors have adequately addressed your comments raised in a previous round of review and you feel that this manuscript is now acceptable for publication, you may indicate that here to bypass the “Comments to the Author” section, enter your conflict of interest statement in the “Confidential to Editor” section, and submit your "Accept" recommendation.

Reviewer #1: All comments have been addressed

**********

2. Is the manuscript technically sound, and do the data support the conclusions?

The manuscript must describe a technically sound piece of scientific research with data that supports the conclusions. Experiments must have been conducted rigorously, with appropriate controls, replication, and sample sizes. The conclusions must be drawn appropriately based on the data presented. 

Reviewer #1: Yes

**********

3. Has the statistical analysis been performed appropriately and rigorously? 

Reviewer #1: Yes

**********

4. Have the authors made all data underlying the findings in their manuscript fully available?

The PLOS Data policy requires authors to make all data underlying the findings described in their manuscript fully available without restriction, with rare exception (please refer to the Data Availability Statement in the manuscript PDF file). The data should be provided as part of the manuscript or its supporting information, or deposited to a public repository. For example, in addition to summary statistics, the data points behind means, medians and variance measures should be available. If there are restrictions on publicly sharing data—e.g. participant privacy or use of data from a third party—those must be specified.

Reviewer #1: Yes

**********

5. Is the manuscript presented in an intelligible fashion and written in standard English?

PLOS ONE does not copyedit accepted manuscripts, so the language in submitted articles must be clear, correct, and unambiguous. Any typographical or grammatical errors should be corrected at revision, so please note any specific errors here.

Reviewer #1: Yes

**********

6. Review Comments to the Author

Please use the space provided to explain your answers to the questions above. You may also include additional comments for the author, including concerns about dual publication, research ethics, or publication ethics. (Please upload your review as an attachment if it exceeds 20,000 characters)

Reviewer #1: Overall the paper has substantially improved. Below are listed my few remaining points that I believe need to be clarified:

1. Pag.1, first paragraph. I think that this phrase is not clear: “An important component of these policies is related to the financial compensation and the duration and height for which it is provided to absent employees”, probably the authors mean:” A key aspect of these policies concerns the financial compensation provided to absent employees, including its amount and the length of time it is offered.

2. Page 9 last paragraph, change into or improve clarity: Our findings indicate that a stated reduction in rewards leads to a higher recovery rate, suggesting an improvement in health conditions that sufficiently facilitates recovery.

3. Pag 10, fifth paragraph: change salaries into wages.

**********

7. PLOS authors have the option to publish the peer review history of their article (what does this mean?). If published, this will include your full peer review and any attached files.

If you choose “no”, your identity will remain anonymous but your review may still be made public.

**Do you want your identity to be public for this peer review?** For information about this choice, including consent withdrawal, please see our Privacy Policy.

Reviewer #1: No

**********

[NOTE: If reviewer comments were submitted as an attachment file, they will be attached to this email and accessible via the submission site. Please log into your account, locate the manuscript record, and check for the action link "View Attachments". If this link does not appear, there are no attachment files.]

While revising your submission, please upload your figure files to the Preflight Analysis and Conversion Engine (PACE) digital diagnostic tool, https://pacev2.apexcovantage.com/. PACE helps ensure that figures meet PLOS requirements. To use PACE, you must first register as a user. Registration is free. Then, login and navigate to the UPLOAD tab, where you will find detailed instructions on how to use the tool. If you encounter any issues or have any questions when using PACE, please email PLOS at figures@plos.org. Please note that Supporting Information files do not need this step.

# Author comment: Author response to Decision Letter 1

**DOI:** 10.1371/journal.pone.0305235.r004

18 May 2024

Dear reviewer,

We have prepared a revised manuscript that incorporates the suggested textual changes. Please find our response in the attached letter.

Attachment Submitted filename: response_letter.pdf

# Editor report: Decision Letter 2

**DOI:** 10.1371/journal.pone.0305235.r005

**Authors:** Paolo Ghinetti

28 May 2024

Effect of financial incentives on the cost and duration of sickness absence.

PONE-D-23-38392R2

Dear Dr. Timp,

We’re pleased to inform you that your manuscript has been judged scientifically suitable for publication and will be formally accepted for publication once it meets all outstanding technical requirements.

Within one week, you’ll receive an e-mail detailing the required amendments. When these have been addressed, you’ll receive a formal acceptance letter and your manuscript will be scheduled for publication.

An invoice will be generated when your article is formally accepted. Please note, if your institution has a publishing partnership with PLOS and your article meets the relevant criteria, all or part of your publication costs will be covered. Please make sure your user information is up-to-date by logging into Editorial Manager at Editorial Manager® and clicking the ‘Update My Information' link at the top of the page. If you have any questions relating to publication charges, please contact our Author Billing department directly at authorbilling@plos.org.

If your institution or institutions have a press office, please notify them about your upcoming paper to help maximize its impact. If they’ll be preparing press materials, please inform our press team as soon as possible -- no later than 48 hours after receiving the formal acceptance. Your manuscript will remain under strict press embargo until 2 pm Eastern Time on the date of publication. For more information, please contact onepress@plos.org.

Kind regards,

Paolo Ghinetti, PhD

Academic Editor

PLOS ONE

Additional Editor Comments (optional):

Reviewers' comments:

# Editor report: Acceptance letter

**DOI:** 10.1371/journal.pone.0305235.r006

**Authors:** Paolo Ghinetti

31 May 2024

PONE-D-23-38392R2 

PLOS ONE

Dear Dr. Timp, 

I'm pleased to inform you that your manuscript has been deemed suitable for publication in PLOS ONE. Congratulations! Your manuscript is now being handed over to our production team.

At this stage, our production department will prepare your paper for publication. This includes ensuring the following:

* All references, tables, and figures are properly cited

* All relevant supporting information is included in the manuscript submission,

* There are no issues that prevent the paper from being properly typeset

If revisions are needed, the production department will contact you directly to resolve them. If no revisions are needed, you will receive an email when the publication date has been set. At this time, we do not offer pre-publication proofs to authors during production of the accepted work. Please keep in mind that we are working through a large volume of accepted articles, so please give us a few weeks to review your paper and let you know the next and final steps. 

Lastly, if your institution or institutions have a press office, please let them know about your upcoming paper now to help maximize its impact. If they'll be preparing press materials, please inform our press team within the next 48 hours. Your manuscript will remain under strict press embargo until 2 pm Eastern Time on the date of publication. For more information, please contact onepress@plos.org.

If we can help with anything else, please email us at customercare@plos.org.

Thank you for submitting your work to PLOS ONE and supporting open access. 

Kind regards, 

PLOS ONE Editorial Office Staff

on behalf of

Professor Paolo Ghinetti 

Academic Editor

PLOS ONE
